# Uncovering the Unseen: *Bordetella hinzii* Emerges in a Lung Transplant Recipient

**DOI:** 10.3390/ijms25094708

**Published:** 2024-04-26

**Authors:** Damiana-Maria Vulturar, Benoît Pilmis, Claire Rouzaud, Anne Gigandon, Gaëlle Dauriat, Séverine Feuillet-Soummer, Liviu-Stefan Moaca, Elie Fadel, Olaf Mercier, Dominique Fabre, Olivier Lortholary, Jérôme Le Pavec

**Affiliations:** 1 Pneumology and Lung Transplantation Department, Marie-Lannelongue–Saint Joseph Hospital Group, 92350 Le Plessis-Robinson, Francej.lepavec@ghpsj.fr (J.L.P.); 2 Department of Pneumology, Iuliu Hatieganu University of Medicine and Pharmacy, 400332 Cluj-Napoca, Romania; 3 Mobile Clinical Microbiology Team, Paris Saint-Joseph Hospital Group, 75014 Paris, France; 4 UMR_1319, Micalis Institute, Paris-Saclay University, INRAe, AgroParisTech, 92290 Châtenay-Malabry, France; 5 Necker Pasteur Centre for Infectious Diseases and Tropical Medicine, IHU Imagine, Necker Enfants Malades, University Hospital, 75015 Paris, France; 6 Institut Pasteur, Université Paris Cité, 75015 Paris, France; 7 Microbiology Department, Marie-Lannelongue–Saint Joseph Hospital Group, 92350 Le Plessis-Robinson, France

**Keywords:** *B. hinzii*, lung transplant, pulmonary infection, resistance

## Abstract

*Bordetella hinzii (B. hinzii)*, a Gram-negative bacillus commonly associated with respiratory infections in animals, has garnered attention for its sporadic cases in humans, particularly in immunocompromised individuals. Despite its opportunistic nature, there remains limited understanding regarding its pathogenicity, diagnostic challenges, and optimal treatment strategies, especially in the context of immunosuppression. Herein, we present the first documented case of acute bronchitis caused by *B. hinzii* in an immunocompromised patient following double-lung transplantation. The patient, a former smoker with sarcoidosis stage IV, underwent transplant surgery and subsequently developed a febrile episode, leading to the identification of *B. hinzii* in broncho-alveolar lavage samples. Antimicrobial susceptibility testing revealed resistance to multiple antibiotics, necessitating tailored treatment adjustments. Our case underscores the importance of heightened awareness among clinicians regarding *B. hinzii* infections and the imperative for further research to elucidate its epidemiology and optimal management strategies, particularly in immunocompromised populations.

## 1. Introduction

*B. hinzii* is a Gram-negative bacillus belonging to the genus *Bordetella*, which also includes other well-known species like *Bordetella pertussis* (causing whooping cough) and *Bordetella bronchiseptica* (causing respiratory infections in animals).

*B. hinzii* has gained attention for its association with respiratory infections in animals, especially birds and rodents. It has been found in a variety of avian species, including poultry, pigeons, and pet birds, and rodents, such as mice and rats, as it was isolated from wild rodents in Southeast Asia; these animals might serve as a reservoir [1,2]. In humans, *B. hinzii* infections have been documented, but they are relatively rare, with just 18 cases reported in the specialty literature [3]. Regarding respiratory infections, it is supposed that the germ colonizes the respiratory airways and then it can cause infection if the host passes through a state of immunocompromise, its activity being similar to that of an opportunistic pathogen. If treated with the correct antibiotics, the patient usually recovers from this infection [4], but there are also cases reported to have led to death [5,6]. Despite its opportunistic nature, there is limited understanding of its pathogenicity, diagnostic challenges, and optimal treatment strategies, particularly in immunocompromised individuals.

The clinical range of activity of the pathogen includes pneumonia, urinary tract infection, bacteriemia, infective endocarditis, liver abscess, or soft tissue abscesses [7,8,9,10].

To the best of our knowledge, we describe the first case of acute bronchitis in an immunocompromised patient following a double-lung transplantation (DLTx) caused by *B. hinzii*. This case not only sheds light on the clinical implications of *B. hinzii* in vulnerable patient populations but also underscores the need for enhanced understanding and vigilance in managing infections caused by this emerging pathogen.

## 2. Case Presentation

In July 2023, a 43-year-old man, a former smoker with a history of sarcoidosis stage IV, who worked as a welder, without any exposure to poultry or other animals, underwent DLTx due to chronic respiratory failure associated with chronic obstructive pulmonary disease (COPD) stage IV, complicated with multiples infectious exacerbations, two pneumothoraxes, and bronchiectasis. Immunosuppression after transplant was maintained with an immunosuppressive regimen of tacrolimus (goal trough of 10–12 ng/mL at the time of this hospitalization), 3000 mg mycophenolic acid, and 15 mg prednisone.

One month following the DLTx surgery, the patient developed a febrile episode and inflammatory markers elevation (C-reactive protein of 45 mg/L, reference range < 5 mg/L). Empiric antibiotic therapy with intravenous cefepime 6g and oral levofloxacine 500 mg bid was initiated due to a history of multiple infectious exacerbations pre-transplantation (*Achromobacter xylosoxydans*, *Stenotrophomonas maltophilia*, *Haemophilus influenzae*, and multidrug resistant *Escherichia coli*).

**Figure 1:**
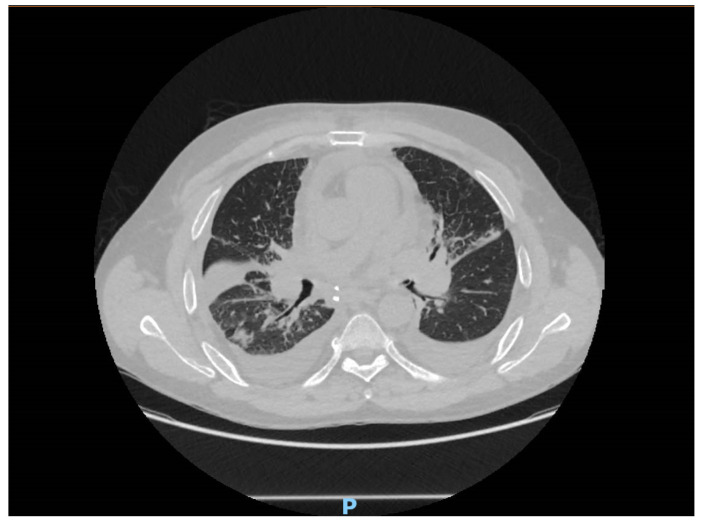
CT scan of the patient.

The computed tomography imaging showed bronchial congestion of the left upper lobar bronchia, with bilateral parenchymal condensations more marked at the level of the middle lobe and the right basal pyramid. Also, there were some areas of ground glass opacities with septal thickening, suggesting a possible infection (Figure 1).

**Figure 2:**
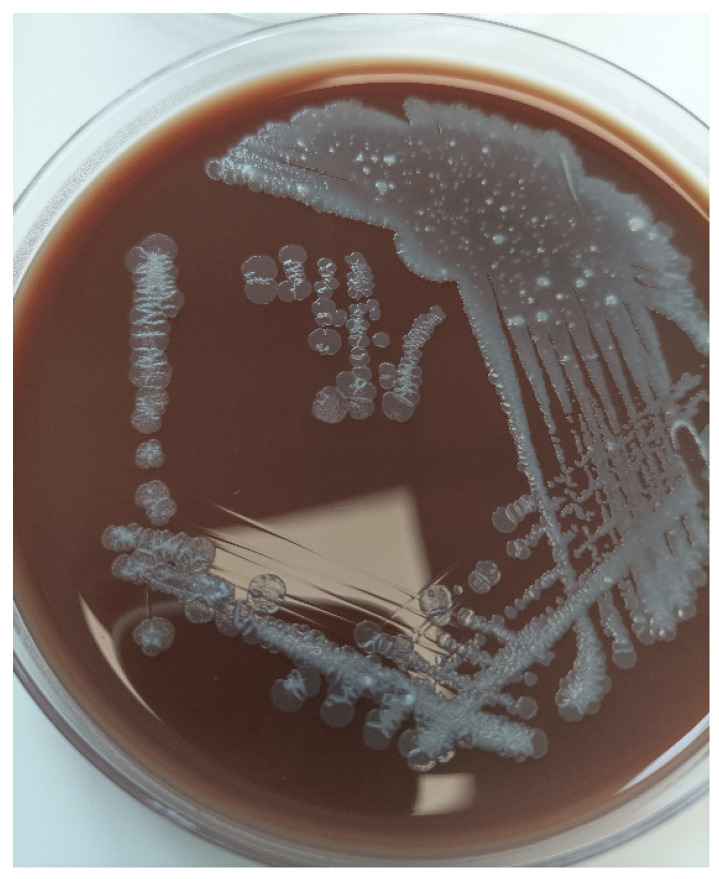
*Bordetella hinzii* colony on horse blood agar.

A broncho-alveolar lavage was performed from the middle lobe and showed 10^7^ CFU/mL *B. hinzii. B. hinzii* grew on horse blood agar (bioMérieux^®^, BioMérieux, Lyon, France) at 37 °C after incubation for 24 h as smooth, grey colonies (Figure 2).

We identified *B. hinzii* using matrix-assisted laser desorption/ionization time-of-flight mass spectrometry (Biotyper Bruker^®^, Bruker Daltonics, Bremen, Germany). We performed antimicrobial susceptibility testing using disk diffusion and interpreted results using the 2021 European Committee on Antimicrobial Susceptibility Testing (nonspecies related) breakpoint. *B. hinzii* showed resistance to amoxicillin, cefotaxime, and aminoglycosides; intermediate resistance to cefepime; and susceptibility to piperacillin/tazobactam, meropenem, imipenem, ertapenem, and trimethoprim/sulfamethoxazole. For colistin, we performed antimicrobial susceptibility testing by using Etest (bioMérieux^®^), and the minimal inhibitory concentration was 2 mg/L (Table 1).

According to the antibiogram results, we switched treatment to piperacillin/tazobactam 4.5 g tid. Subsequent to the persistence of symptoms, the patient underwent a second bronchial aspiration procedure 7 days later, which resulted in the confirmation of *B hinzii* infection through diagnostic testing. New antibiotic susceptibility tests were carried out, identifying acquired resistance to piperacillin/tazobactam and a susceptibility to carbapenems, trimethoprim/sulfamethoxazole, and doxycycline. In this context, the antibiotic therapy was switched to doxycycline 100 mg tid for 14 days and inhalative antibiotic treatment with colistin (2 MUI twice a day).

Based on the post operative clinical status, the patient was secondarily diagnosed with a grade I acute cellular reject and treated with a bolus of Solumedrol 1 g/day for three days. However, despite the adapted treatment for *B. hinzii*, the patient remained positive for the sprout, leading to a delayed treatment of the acute cellular rejection. The treatment for *B. hinzii*, based on oral doxycycline and nebulised colistin, was then prolonged for 14 additional days, leading to negative pulmonary samples after one month.

## 3. Discussion

Initially deemed harmless in birds, *B. hinzii*, a Gram-negative bacillus causing respiratory illnesses in poultry, has shown potential pathogenicity in some veterinary isolates. It sporadically appears in rabbits and rodents, causing pulmonary issues in mice and bloodstream infections in rats [2,11,12]. Human cases, rare and often associated with immunosuppression, SARS-CoV-2 infection, or prior poultry exposure, manifest as diverse infections, including pneumonia with or without bacteraemia. Respiratory tract infections present symptoms like cough, runny nose, congestion, and fever, with severity depending on the *Bordetella* species and individual immune status [4]. Despite our patient’s lack of exposure to poultry or birds, an increasing number of reported cases suggest a growing awareness of *B. hinzii* infections, possibly due to the pathogen’s emergence, improved identification methods, and an expanding population at risk, particularly the immunosuppressed. In our case, the bacteria were resistant to amoxicillin, cefotaxime, and aminoglycosides, and intermediate-resistant to cefepime; and they acquired resistance to piperacillin/tazobactam during the treatment. Reported *B. hinzii* isolates were frequently multidrug-resistant, including resistance to cephalosporins, aminoglycosides, and quinolones, but remained susceptible to piperacillin/tazobactam, carbapenems, and cyclines. The interpretation of antimicrobial susceptibility testing is not established, and the choice of antimicrobial drugs and treatment duration are not standardized.

There are case reports published in the literature regarding other *B. hinzii* infection in organ transplant patients, but no other published case of *B. hinzii* in a lung transplant recipient. Pechacek et al. describe an unusual case of meningitis caused by *B. hinzii* in a patient with kidney transplant history. In this study, the clinical isolates of *B. hinzii* displayed a susceptibility pattern to various antibiotics. They were found to be sensitive to carbapenems, piperacillin, and trimethoprim/sulfamethoxazole. However, their sensitivity to ciprofloxacin was only intermediate, suggesting that while ciprofloxacin could potentially be effective against the infection, it may not be the ideal or first-choice antibiotic due to this intermediate sensitivity [13]. Furthermore, Arvand et al. described in his article a patient with a medical background including several liver transplantations. The patient experienced recurring episodes of cholangitis, and over a span of six months, *B. hinzii* was consistently found in various samples taken from the biliary tract. While deciphering laboratory and histological results can be challenging for individuals who have undergone liver transplantation due to primary sclerosing cholangitis, it was posited that *B. hinzii* played a role as the causative agent behind the prolonged biliary duct infection in this patient. Also, the bacteria resistance to β-lactam and fluoroquinolones was noted [10].

Another study by Fabre et al. showed an infection of *B. hinzii* in a bone marrow transplantation patient, and described the potential of the pathogen to spread from an avian reservoir [4].

In our case, the patient was positive for almost one month, but there are some studies that provided evidence of the extended presence of *B. hinzii* in the respiratory and gastrointestinal systems [9,10]. *B. hinzii* manifests as an opportunistic pathogen, leading to respiratory infections in individuals with cystic fibrosis [14]. During a 12-month period, it was detected on eight separate occasions in the respiratory system of a cystic fibrosis patient [9]. However, the infection with *B. hinzii* could be one of the potential factors involved in the development of the cellular reject taking into consideration the resistance of the bacteria despite of the right treatment.

## 4. Conclusions

In conclusion, this is the first reported case of *B. hinzii* pulmonary infection in a lung transplant patient. While the exact transmission route remains unclear, the potential association with occupational exposure to *B. hinzii* in the respiratory tract cannot be definitively affirmed. While our findings highlight the emergence of this pathogen as a potential cause of pulmonary infections post transplantation, several important questions remain unanswered. The exact transmission route of Bordetella hinzii in immunocompromised individuals, particularly during the COVID-19 pandemic, warrants further investigation.

Moreover, the challenges encountered in diagnosing and treating *B. hinzii* infections underscore the importance of continued research efforts aimed at elucidating the epidemiology, pathogenesis, and optimal management approaches for this emerging pathogen. Given the rarity of reported cases and the diverse clinical manifestations associated with *B. hinzii* infections, collaborative multicentre studies may offer valuable insights into the prevalence, risk factors, and outcomes of such infections in different patient populations.

Furthermore, considering the potential association between occupational exposure and the presence of *B. hinzii* in the pulmonary tract, future studies should explore preventive measures and infection control strategies, especially in high-risk settings such as healthcare facilities and animal-related occupations. Finally, the increasing recognition of *B. hinzii* infections underscores the importance of vigilance among clinicians and microbiologists in identifying and managing these infections, particularly in immunocompromised individuals.

In summary, this case report underscores the need for continued research efforts to enhance our understanding of Bordetella hinzii infections and to develop evidence-based guidelines for their diagnosis, treatment, and prevention in transplant recipients and other vulnerable patient populations.

## Figures and Tables

**Table 1:** Antibiotic susceptibility test results.

Antibiotic	*Bordetella hinzii* First Isolate			*Bordetella hinzii* Second Isolate		
	Susceptibility Interpretation	Zone Diameter Inhibition (mm)	MIC Values (mg/L)	Susceptibility Interpretation	Zone Diameter Inhibition (mm)	MIC Values (mg/L)
**Amoxicillin**	Resistant	6	-	Resistant	6	-
**Amoxicillin-clavulanate**	Resistant	6	-	Resistant	6	-
**Piperacillin-tazobactam**	Susceptible	29	-	Susceptible	31	-
**Cefotaxim**	Resistant	6	-	Resistant	6	-
**Ceftazidim**	Resistant	15	-	Resistant	6	-
**Cefepime**	Intermediate	22	-	-	-	-
**Ertapenem**	Susceptible	34	-	-	-	-
**Imipenem**	Susceptible	29	-	Susceptible	32	-
**Gentamicin**	Resistant	8	-	Resistant	6	-
**Trimethoprim-sulfamethoxazole**	Susceptible	42	-	Susceptible	25	-
**Colistine**	-	-	-	Susceptible	-	2

## Data Availability

Data is contained within the article.
